# Impact of COVID-19 pandemic on renal care services in Nigeria

**DOI:** 10.11604/pamj.supp.2020.35.24403

**Published:** 2020-07-04

**Authors:** Oluseyi Ademola Adejumo

**Affiliations:** 1 Department Of Internal Medicine, University of Medical Sciences, Ondo State

**Keywords:** Chronic kidney disease, COVID-19, pandemic, renal care, Nigeria

## Abstract

The COVID-19 pandemic has altered the course of events globally. Enforcement of lock down orders to curtail the spread of the pandemic had untoward consequences on the economy and health of the citizenry. In Nigeria, access to renal care was reduced by restriction of movement; inability to afford care due to economic downturn; suspension of transplant programs; uncertainties about dialysis guidelines; anxiety and reduced motivation of health care workers (HCWs) due to lack of government’s commitment to their welfare and increasing rate of COVID-19 infection among HCWs. Formulation and implementation of policies to improve HCWs welfare and ease the burden of CKD patients should be prioritized in order to ensure optimal care of renal patients during the present pandemic.

## Commentary

The coronavirus disease-19 (COVID-19) pandemic has altered the course of events globally since its outbreak in late 2019. Globally, over 8,184,867 cases and 443,872 deaths have been recorded across the world as at 18th June, 2020 [1]. The first incident of the virus was recorded in Nigeria in February 2020 and a total of 17,735 cases and 469 deaths have been reported [2]. The United Nations Development Programme (UNDP) reported that the pandemic will place huge pressure on Nigeria´s already fragile healthcare system which has been further worsened by the exodus of medical practitioners from the country in recent times [3]. The socio-economic, physical and psychological burden of chronic kidney disease (CKD) is high especially in developing countries like Nigeria where majority of those affected are in the productive age group and rather become an economic burden to their families [4,5]. The ongoing pandemic had devastating effect on CKD patients especially during the peak when the government had to ensure total lock down. The lock down grounded economic activities in the country and severely limited movement of people in order to curtail the spread of the disease. However, this was not without adverse consequences on health care of those with chronic medical condition such as CKD.

Chronic kidney disease patients are at higher risk of having severe COVID-19 and succumbing to the disease due to higher prevalence of cardiovascular risks such as diabetes, hypertension, hypoalbuminaemia and anaemia compared to the general population [6]. Also, reduced immunity from steroid and immunosuppressant therapy especially in transplant patients contribute to high severity of COVID-19 and associated mortality. Accessibility of end stage renal disease patients to dialysis during the peak of the COVID-19 pandemic in Nigeria was reduced because of the adverse economic effect of the total lock down in some states since majority paid out of pocket to finance their care. Restriction of movement during the total lock down period also affected the timely transportation of patients to their dialysis centres even in cases where funding was not a major challenge. The impracticability of renal patients on maintenance dialysis to totally comply with the lock down order and social distancing further exposes them to risk of getting infected with SARS-CoV-2.

Majority of renal transplant programmes were suspended therefore denying renal patients who could afford transplant, better quality of life. The suspension was due to uncertainties such as fear of shortage of personnel to monitor patients especially in the early postoperative period; increased susceptibility to COVID-19 due to high dose of immunosuppressants during the induction period of transplant; issues with methodology and sensitivity of COVID-19 testing that may lead to inadvertent transplant of donor kidneys infected with SARS-CoV-2 [7,8]. The travel ban and airport closures across the world also posed a challenge for patients who were already billed for transplant outside the country. Medical tourism especially for CKD patients has been a major issue in Nigeria. This is owing to the fact that there are limited health care facilities with capacity, personnel and experience to carry out transplant; lack of political will by government to fund transplant programmes; high cost of procedure and lack of healthcare insurance to cover cost [9]. Such patients have had to wait endlessly for the lockdown of airports to be eased before they can travel for their medical care.

The rate of infection of health care workers (HCWs) with COVID-19 across the world and particularly, the increasing rate of infection of HCWs in the African region is of concern. Number of infected HCWs has consistently increased in Nigeria which has recorded the highest number of infected HCWs in the African region in the last few weeks. HCWs in renal centers are thus concerned about risk of transmission of COVID-19 to them by renal patients during treatment especially with limited supplies of personal protective equipment (PPE). The morale of HCWs in Nigeria to fight the COVID-19 pandemic is dampened because the government had not shown enough commitment to their welfare. Presently, the monthly hazard allowance of HCWs is less than 15USD and majority are not under life insurance cover by the government. The payment of the newly approved hazard allowance that was occasioned by the COVID-19 pandemic is still yet to be implemented.

There were also uncertainties about guidelines that suit renal units in order to protect other kidney disease patients and HCWs while attending to patients with COVID-19 requiring dialysis. The recently published guidelines for the prevention, detection and management of the renal complications of COVID-19 by the African Association of Nephrologists has been able to clarify gray areas and douse anxiety among renal health care workers [10]. This was a step in the right direction as the guidelines took cognizance of the peculiarities of the African setting as well as the role of all major stakeholders in renal care. Despite the guidelines, HCWs in renal units have to take necessary additional precautions and wear full PPEs for long periods while monitoring renal patients on dialysis; hence they feel they deserve additional remuneration as frontline workers.

## Conclusion

It is evident and highly imperative that both Nigerian government and corporate organizations need to show greater and genuine commitment to the welfare of the HCWs especially those on the frontline in this present pandemic. Policy formulation and implementation that will ease the burden of CKD patients should be prioritized in order to ensure optimal care delivery to renal patients during this present pandemic.
